# The contribution of parent and youth information to identify mental health disorders or problems in adolescents

**DOI:** 10.1186/s13034-017-0160-9

**Published:** 2017-04-28

**Authors:** Marcel Aebi, Christine Kuhn, Tobias Banaschewski, Yvonne Grimmer, Luise Poustka, Hans-Christoph Steinhausen, Robert Goodman

**Affiliations:** 1 Department of Child and Adolescent Psychiatry, University Hospital of Psychiatry Zurich, Zurich, Switzerland; 2 Department of Forensic Psychiatry, University Hospital of Psychiatry Zurich, Neptunstrasse 60, 8032 Zurich, Switzerland; 3 Department of Psychology, Clinical Psychology for Children/Adolescents and Couples/Families, University of Zurich, Zurich, Switzerland; 4 Department of Child and Adolescent Psychiatry and Psychotherapy, Central Institute of Mental Health, Medical Faculty Mannheim, University of Heidelberg, Heidelberg, Germany; 5 Department of Child and Adolescent Psychiatry/Psychotherapy, University of Göttingen, Göttingen, Germany; 6 Child and Adolescent Mental Health Centre, Capital Region Psychiatry, Copenhagen, Denmark; 7 Clinical Psychology and Epidemiology, Department of Psychology, University of Basel, Basel, Switzerland; 8 Department of Child and Adolescent Psychiatry, King’s College London Institute of Psychology, Psychiatry & Neuroscience, London, UK

**Keywords:** Adolescent psychopathology, Emotional problems, Behavioural problems, Multi-informants, SDQ, DAWBA

## Abstract

**Background:**

Discrepancies between multiple informants often create considerable uncertainties in delivering services to youth. The present study assessed the ability of the parent and youth scales of the Strength and Difficulties Questionnaire (SDQ) to predict mental health problems/disorders across several mental health domains as validated against two contrasting indices of validity for psychopathology derived from the Development and Well Being Assessment (DAWBA): (1) an empirically derived computer algorithm and (2) expert based ICD-10 diagnoses.

**Methods:**

Ordinal and logistic regressions were used to predict any problems/disorders, emotional problems/disorders and behavioural problems/disorders in a community sample (n = 252) and in a clinic sample (n = 95).

**Results:**

The findings were strikingly similar in both samples. Parent and youth SDQ scales were related to any problem/disorder. Youth SDQ symptom and impact had the strongest association with emotional problems/disorder and parent SDQ symptom score were most strongly related to behavioural problems/disorders. Both the SDQ total and the impact scores significantly predicted emotional problems/disorders in males whereas this was the case only for the total SDQ score in females.

**Conclusion:**

The present study confirms and expands previous findings on parent and youth informant validity. Clinicians should include both parent and youth for identifying any mental health problems/disorders, youth information for detecting emotional problems/disorders, and parent information to detect behavioural problems/disorders. Not only symptom scores but also impact measures may be useful to detect emotional problems/disorders, particularly in male youth.

**Electronic supplementary material:**

The online version of this article (doi:10.1186/s13034-017-0160-9) contains supplementary material, which is available to authorized users.

## Background

Youth and parent screening measures such as the Strength and Difficulties Questionnaire [SDQ; 1, 2] or the Achenbach Systems of Empirically Based Assessments [ASEBA; 3] are easy to use and cost-effective methods to identify adolescents with psychological difficulties. Both of these instruments are highly popular among mental health practitioners and researchers and also among other child care professionals. They have been translated into many different languages and implemented in clinical processes worldwide. Mental health professionals use these screening measures to decide whether further and more detailed assessments of emotional or behavioural disorders are indicated. Researchers use these screening measures in epidemiological and clinical studies to measure the type, the extent, and the course of mental health problems. Nurses and practitioners in general hospitals and social workers in schools and juvenile justice institutions use these screening measures to decide which adolescents need more specific assessment and treatment and should be referred to mental health practitioners. However, discrepancies between multiple informants often create considerable uncertainties in delivering services to youth and drawing conclusions from research [4].

Informant discrepancies on mental health problems are one of the major challenges in child and adolescent psychiatry. A recent meta-analysis of 341 studies [5] found that modest cross-informant agreement is one of the most robust phenomena in clinical child and adolescent research (with mean correlation: r = 0.28). However, the degree of cross-informant agreement for mental disorders varies between mental health domains, different societies and cultures and also depends on the youth’s age and gender [5–8].

A number of different factors contribute to informant discrepancies on mental health problems [9, 10]. First, some mental health problems emerge only in specific situations such as school and family contexts or within peer interactions. Contextual variations occur within a variety of psychiatric domains including social anxiety, attention-deficit-hyperactivity, and conduct problems [e.g., 11–13]. Secondly, informants (e.g., parent and youth) may differ on their perceptions and awareness of mental health problems and what kinds of behaviours are within the norm. For example, parents may be worried about the adolescent’s withdrawal, whereas the adolescent perceives his behaviour as within the normal range and views the intrusiveness of the parents as the area of concern. Thirdly, informant discrepancies may result from measurement errors in regard to the frequency and severity of behavioural, emotional or hyperactivity problems.

Different strategies have been suggested for how to choose informants and how to aggregate data from multiple informant data for diagnostic decision making [12, 14]. In order to disentangle three meaningful components of psychopathology such as (1) the trait (measure of interest for youth’s psychopathology), (2) the context (factors related to the emergence and the reporting of symptoms), and (3) the informants perspective, principal component analysis and regression analyses have been proposed [15, 16]. However, these approaches are quite complex and cannot easily be implemented into clinical practice.

Two factors seem crucial for researchers and clinicians to decide whether parent or youth information is more accurate: (1) the area of mental health problems addressed (e.g., emotional vs. behavioural problems) and (2) the context in which the assessment took place (e.g., clinical vs. community assessments) [17, 18]. For detecting any mental health problems, information from both informants can be useful [19]. In a community sample, parent and youth information uniquely and indispensably contributed to later signs of maladjustment (referral to mental health services, need for professional help, and presence of a disorder) [20]. Similarly, both, self-reports and parent reports were found necessary to detect the presence of a psychiatric diagnosis in a clinical outpatient sample [17].

To explore emotional problems/disorders such as depression and anxiety, clinicians and researchers usually rely on adolescents’ self-reports from questionnaires or interviews because adolescents themselves are assumed to be the most valid source of information for this kind of problems [21]. In fact, adolescents do report significantly more internalizing symptoms than their parents in clinical samples [22, 23] and community samples [24]. Furthermore, self-information has been found accurate to predict the presence of internalizing problems/emotional disorders in community as well as in clinical samples [8, 17, 20, 21, 25–27]. However, some studies also found that the inclusion of parent information further increased the ability to detect emotional problems in community and clinical samples [17, 28].

In the exploration of externalizing problems such as attention-deficit-hyperactivity disorder (ADHD), oppositional defiant disorder (ODD), and conduct disorder (CD), parent information has been considered to be more valid than youth self-reports by mental health professionals [21]. Though on theoretical grounds, self-reports also seem important to assess conduct problems, because many of these behaviours (e.g., thefts, fire setting, physical attacks) occur in setting to which parents are not privy [22]. In community samples, adolescent self-reports show higher levels of behavioural problems than parents reports [18, 24] and adolescent self-reports were found to be valid predictors of externalizing problems, behavioural disorders and later criminal behaviours [20, 28–31]. In clinical samples, adolescents may underreport behavioural problems [18, 32] and adolescent self-reports are sometimes less accurate than parent reports in detecting behavioural disorders [17]. Some adolescents may minimize their conduct problems to avoid possible adverse consequences of full disclosure [33].

Previous studies testing the informant validity of parent and adolescent self-ratings reported conflicting findings and were limited by the use of either just community or just clinical samples and by a paucity of validation measures, (e.g., relying on clinicians’ diagnoses of unclear reliability). Furthermore, previous studies did not consider impact measures as additional information to detect psychiatric disorders. Some adolescents find it hard to report psychological symptoms and may find it easier to describe specific impairments in school, family and peer group. Given the previous findings on the validity of the SDQ impact scales [34], we predicted that impact measures in addition to symptoms scores would make a useful contribution to the assessment of mental health disorders.

The present study intended to confirm and expand previous findings by analysing data collected in a community and an outpatient sample. The ability of parent and youth SDQ scales measuring problems and impact were analysed in order to predict mental health problems/disorders across several mental health domains (any disorder, emotional disorders, behavioural disorders), as validated against two contrasting indices of validity derived from the Development and Well-Being Assessment, DAWBA (see method section below): One approach used the empirically developed multi-informant DAWBA bands (ordinal measures) based on a computer algorithm to aggregate parent and/or youth information from structured interview questions, while the other approach used ICD-10 diagnosis generated by expert DAWBA raters, i.e., experienced clinicians who rated the presence of an ICD-10 disorders after reviewing the answers to closed and open-ended questions. Because the DAWBA is a well validated multi-informant based instrument [35, 36], the current study may overcome some methodological limitations of diagnoses derived from single informants or unstructured clinical evaluations.

Based on the existing literature, we hypothesized that in multivariate analyses (1) the *youth and parent* SDQ total scores would both be highly associated with any problems/disorders in both samples, (2) the *youth* SDQ total score would be more strongly associated with emotional problems/disorders than the *parent* SDQ total score in both samples, (3) *parent and youth* SDQ total scores would be associated with behavioural problems/disorders in the community sample, (4) but *only parent* SDQ total score would be associated with behavioural problems/disorders in the clinical sample. Hypotheses 3 and 4 were established a posteriori in accordance with findings from previous studies. We further assumed that youth and parent SDQ impact scores would supplement the predictive power of symptoms scores in the prediction of any problems/disorders, emotional problems/disorders, and behavioural problems/disorders in both samples.

In addition, we tested the ability of the SDQ conduct and emotional problem scales in the prediction of emotional and behavioural problems/disorders in both samples. Further supplemental analyses of parent and youth SDQ hyperactivity and conduct problem scales in the prediction of ODD, CD and ADHD were performed in the clinic sample only (because of the low prevalence rates of these disorders in the community sample).

## Methods

### Samples

The present study is based on a community and clinic sample from two different sites [19]. The community sample is one arm of the IMAGEN study described in more detail in [37]. A sample of healthy adolescents was recruited from secondary schools in the city of Mannheim, Germany, and surrounding areas via flyers, school visits and residents’ registration offices. The recruitment was based on two criteria: (1) Greatest possible diversity in terms of socio-economic status, cognitive and emotional development. To achieve this goal, private- and state-funded schools and special educational schools (classes) were equally targeted; (2) Minimization of the ethnic heterogeneity by selecting a sample of young people with European ethnicity. Exclusion criteria were severe complications during pregnancy and birth, serious pre-existing conditions, (particularly neurological and psychiatric disorders), IQ < 70 and contraindications for a parallel magnetic resonance imaging study, e.g., severe claustrophobia or metal/electronical implants [37]. The study was approved by the local ethics committee of the University of Mannheim. The final community sample consisted of 252 adolescents (46.8% male) with a mean age of 13.98 years (SD = 0.60 years, range 13–17 years).

The outpatient sample was recruited from all available patients who attended the outpatient centres of the child and adolescent psychiatry service of the canton of Zurich, Switzerland, between September 2007 and June 2009 (n = 875). Out of this sample, 345 youth and parents with sufficient German language skills participated (participation rate = 40.5%). However, only patients aged 11–17 years with available parent and youth information were considered for the present study. There were no further exclusion criteria [35]. The final outpatient sample consisted of 95 patients (66.3% male) with a mean age of 13.95 years (SD = 2.04 years, range 11–17 years). Subjects in both the community and clinical samples were first assessed with the internet-based parent and youth versions of the SDQ [2, 38] and then filled in the online version of the Development and Well-Being Assessment [DAWBA; 36].

### Measures

#### Strength and Difficulties Questionnaire (SDQ)

The SDQ is a questionnaire covering common mental health problems in children aged 2–17. The 20 items relating to emotional symptoms, conduct problems, hyperactivity and peer problems can be summed to generate a total difficulty score ranging from 0 to 40. The SDQ has been shown to have dimensional as well as categorical qualities [1]. The SDQ is commonly administered with an impact supplement that asks whether the respondent thinks the youth has significant difficulties, and if so inquires about overall distress and social impairment—forming the basis for an impact score. In this study, the parent and self-report versions of the SDQ with impact supplement was administered to parents and to youths aged 11 or older and used as a screening measure to predict DAWBA bands/expert ratings across multiple mental health domains. The psychometric properties of the SDQ are well established [1, 39] so that we did not compute them again in the present study.

#### Development and Well-Being Assessment (DAWBA)

The DAWBA [36] includes structured interview sections covering the major mental disorders, followed by a semi-structured part eliciting open-ended descriptions from respondents about areas of concern. Diagnostic predictions in line with ICD-10 and DSM-IV criteria can be generated by computerized algorithms drawing on data from the structured questions, generating what are called “DAWBA bands” [40]. The DAWBA bands are based on an algorithm that combines the information from symptom and impact measures from all available respondents, e.g., parent report and adolescent report. It is not an average or an addition, but aims to follow the logic of the DSM and ICD classifications, e.g., giving more weight to symptoms of hyperactivity if reported across different situations and accompanied by impairment. The DAWBA bands algorithm does not prioritise any one category of informant a priori. DAWBA bands have been previously validated in two large samples of British (n = 7912) and Norwegian youth (n = 1364) [40]. In the present study we use the “any disorder” DAWBA band, the emotional disorder DAWBA band (affective and anxiety problems) and the behavioural disorder DAWBA band. Supplemental analysis also included specific DAWBA bands for ADHD, ODD, and CD) Since the DAWBA bands are quick, cheap and standardized [40], they have been used as the only source of diagnostic ratings in some research studies [e.g., 41]. The DAWBA bands are used as ordinal outcome measures in the present study (frequencies of the probability to meet criteria of a disorder: <0.5%, ~3%, ~15%, ~50%, 70%+). In addition, dichotomous (present versus absent) ratings of ICD-10 disorders (emotional, behavioural, ADHD, CD and ODD) were generated by expert clinicians based on a review of all available information, including open-ended comments. The inter-rater reliability for expert based diagnosis was found to be good (kappa 0.79–0.89) [35].

### Statistical analyses

We used multivariate ordinal and logistic regression to predict total, emotional, and behavioural DAWBA bands (problems) and expert diagnoses (disorders). Besides z-transformed SDQ youth and parent symptom and impact scores we included youth’s age and male gender (males = 1, females = 0) as covariates in the analyses. Because of the small number of psychiatric disorders in the community sample, Firth’s bias-reduced logistic regressions by the use of the package “logistf” [42] in R statistical software were performed [43]. This method is accurate for logistic regression analyses with rare outcome data. None of the linear predictors/covariates showed multicollinearity and the assumption of proportional odds was met for all ordinal regression analyses (χ^2^ > 0.05). In addition, sex-specific receiver operating characteristic (ROC) analyses of SDQ total and impact scores were performed to predict DAWBA expert rated emotional disorders. All analyses were conducted using R statistical software [43] and SPSS 23 for Mac OS X, were two-tailed, and utilized a threshold for statistical significance of p = 0.05.

## Results

Frequencies of the DAWBA bands of the 252 adolescents of the community and the 95 adolescents of the clinic sample are shown in Table 1. As expected and in contrast to the clinical sample, most adolescents from the community sample showed low probabilities for having a mental health disorder according to DAWBA expert ratings (e.g., 3% and less, Table 1). In the community sample 21 (8.3%) adolescents had any ICD-10 disorder, 6 (2.4%) any emotional disorder, 9 (3.6%) any behavioural disorder (ODD 1, 0.4%; CD 8, 3.2%), and 6 (2.4%) any hyperkinetic disorder. In the clinic sample 67 (70.5%) adolescents had any ICD-10 disorder, 41 (43.2%) any emotional disorder, 21 (22.1%) any behavioural disorder (ODD 13, 13.7%; CD 8, 8.4%), and 13 (13.7%) any hyperkinetic disorder. Bivariate correlations of DAWBA bands and disorders (expert diagnosis) in the community and clinical samples are shown in Table 2. All correlations were in the medium range and highly significant in both samples. Bivariate correlations between parent and youth SDQ scores and subscales in the community and the clinical sample are presented in Table 3. With the exception of the SDQ total score and SDQ impact in the clinic sample, all correlations were in the medium range and highly significant in both samples.

**Table 1:** Frequencies of probands in the community (n = 252) and the clinic sample (n = 95) according to probability of having any disorder, any emotional, any behavioural disorder, ADHD, CD and ODD (DAWBA bands)

DAWBA bands	“Any DAWBA”		Emotional DAWBA		Behavioural DAWBA		ADHD DAWBA		CD DAWBA		ODD DAWBA	
Probability of having a disorder	Community	Clinic	Community	Clinic	Community	Clinic	Community	Clinic	Community	Clinic	Community	Clinic
<0.5%	90 (35.7%)	3 (3.2%)	216 (85.7%)	33 (34.8%)	164 (65.1%)	36 (37.9%)	239 (95.2%)	49 (51.6%)	235 (93.3%)	62 (65.3%)	168 (66.7%)	41 (43.2%)
~3%	115 (45.6%)	17 (17.9%)	20 (7.9%)	17 (17.9%)	63 (25.0%)	15 (15.8%)	8 (3.2%)	26 (27.4%)	1 (0.4%)	3 (3.2%)	68 (27.0%)	19 (20.0%)
~15%	30 (11.9%)	26 (27.4%)	14 (5.9%)	20 (21.1%)	14 (5.6%)	16 (16.8%)	4 (1.6%)	16 (16.8%)	6 (2.4%)	16 (16.8%)	11 (4.4%)	10 (10.5%)
~50%	13 (5.2%)	23 (24.2%)	2 (8.0%)	22 (23.2%)	7 (2.8%)	9 (9.5%)	1 (0.4%)	0 (0.0%)	6 (2.4%)	3 (3.2%)	4 (1.6%)	13 (13.7%)
70%+	4 (1.6%)	26 (27.4%)	0 (0.0%)	3 (3.2%)	4 (1.6%)	19 (20.0%)	0 (0.0%)	4 (4.2%)	4 (1.6%)	11 (11.6%)	1 (0.4%)	12 (12.6%)

*DAWBA* Development and Well-being Assessment

**Table 2:** Bivariate correlations of DAWBA bands and corresponding disorders (expert diagnosis) in the community (n = 252) and the clinic sample (N = 95)

	Community sample	Clinic sample
Any problem/disorders	0.62***	0.53***
Emotional problem/disorders	0.31***	0.67***
Behavioural problem/disorders	0.59***	0.60***

*** Significance (two sided), p < .001

**Table 3:** Bivariate correlations of SDQ parent and youth scales in the community (n = 252) and the clinic sample (n = 95)

	Community sample	Clinic sample
SDQ total score	0.46***	0.20 n.s.
SDQ impact	0.45***	0.04 n.s.
SDQ emotion problems	0.36***	0.42***
SDQ behaviour problems	0.38***	0.37***
SDQ hyperactivity	0.49***	0.47***

* Significance (two sided), p < .05, ** significance (two sided), p < .01, *** significance (two sided), p < .001

### Findings in the community sample

Multivariate ordinal and Firth’s bias reduced logistic regressions with DAWBA bands (problems) and expert diagnoses (disorders) as outcome variables are presented in Table 4 and show that the parent SDQ total score (but not the impact score) was related to any problems and disorders, any behavioural problems and disorders, but not to any emotional problems or disorders. The youth SDQ total score was associated with any problems as well as to emotional problems and disorders. The youth SDQ impact score was related to any problems and disorders as well as to emotional problems. Among the SDQ subscales, the parent SDQ emotional problems scale was associated with emotional problems but not with emotional disorders, whereas the youth SDQ emotional problems scale was associated with emotional problems and disorders. The parent but not the youth SDQ behaviour problems subscale was related to any behaviour problems and disorders. Among the covariates, age was negatively related to the presence of an emotional disorder (coefficient = −2.54, 95% CI −4.97 to −0.71). Data of the clinic and community sample is provided in Additional file 1.

**Table 4:** Ordinal regressions and Firth’s biased reduced logistic regressions with SDQ parent and youth measures as predictors of DAWBA bands/disorders in the community sample (N = 252)

	Any problem/disorders		Emotional problem/disorders		Behavioural problem/disorders	
	DAWBA band Estimate (95% CI)	Expert diagn. OR (95% CI)	DAWBA band Estimate (95% CI)	Expert diagn. OR (95% CI)	DAWBA band Estimate (95% CI)	Expert diagn. OR (95% CI)
SDQ total/impact score						
Parent SDQ total score	0.67 (0.34–1.01)***	0.69 (0.11–1.27)*	0.31 (−0.15 to 0.78)	−0.78 (−3.20 to 0.32)	0.77 (0.42–1.12)***	0.93 (0.20–1.70)*
Parent SDQ impact	0.27 (−0.05 to 0.59)	0.12 (−0.33 to 0.57)	−0.25 (−0.73 to 0.23)	0.47 (−0.65 to 2.05)	0.31 (−0.01 to 0.63)	−0.11 (−0.94 to 0.55)
Youth SDQ total score	0.49 (0.19–0.78)**	0.54 (−0.04 to 1.14)	0.62 (0.18–1.06)**	1.51 (0.35–3.25)*	0.14 (−0.18 to 0.46)	0.08 (−0.74 to 0.84)
Youth SDQ impact	0.62 (0.30–0.94)***	0.65 (0.21–1.16)**	0.45 (0.13–0.77)**	0.51 (−0.11 to 1.19)	0.17 (−0.11 to 0.48)	0.06 (−0.44 to 0.51)
SDQ subscales						
Parent SDQ emotion problems	–	–	0.43 (0.10–0.76)*	0.11 (−0.59 to 0.81)	–	–
Youth SDQ emotion problems	–	–	0.89 (0.49–1.30)***	1.22 (0.45–2.19)**	–	–
SDQ subscales						
Parent SDQ behaviour problems	–	–	–	–	1.01 (0.78–1.30)***	1.11 (0.51–1.82)***
Youth SDQ behaviour problems	–	–	–	–	0.26 (−0.02 to 0.55)	0.46 (−0.20 to 1.14)

Age and male gender was included as covariates in the analyses

*SDQ* Strengths and Difficulties Questionnaire, *DAWBA* Development and Well-being Assessment, *OR* odds ratio

* Significance (two sided), p < .05, ** significance (two sided), p < .01, *** significance (two sided), p < .001

### Findings in the clinic sample

Findings from multivariate ordinal and logistic regressions with DAWBA bands (problems) and expert diagnoses (disorders) as outcome variables are presented in Table 5. The parent SDQ total score (but not the impact score) was related to any problems as well as to behavioural problems and disorders. The youth SDQ total score was associated with any problems and disorders as well as with emotional disorders. The youth SDQ impact score was related to emotional problems. The SDQ emotional problems subscales were related to emotional problems and disorders, particularly in the youth report, and to a lesser degree in the parent report. The parent SDQ behaviour problems subscale was associated with behavioural problems and disorders. The youth SDQ behaviour problem subscale was related to a lesser degree than the parent SDQ behaviour problems scale to behavioural problems only. Among the covariates female gender was significantly associated with the presence of an emotional disorder (OR 2.90, 95% CI 1.05–8.05) and male gender with the presence of a behavioural disorders (OR 0.12, 95% CI 0.02–0.66).

**Table 5:** Ordinal and logistic regressions with SDQ parent and youth measures as predictors of DAWBA bands/disorders in the clinical sample (N = 95)

	Any problem/disorders		Emotional problem/disorders		Behavioural problem/disorders	
	DAWBA band Estimate (95% CI)	Expert diagn. OR (95% CI)	DAWBA band Estimate (95% CI)	Expert diagn. OR (95% CI)	DAWBA band Estimate (95% CI)	Expert diagn. OR (95% CI)
SDQ total/impact score						
Parent SDQ total score	1.02 (0.53–1.51)***	1.65 (0.89–3.07)	0.21 (−0.23 to 0.63)	0.72 (0.42–1.23)	0.81 (0.36–1.25)***	3.09 (1.58–6.04)**
Parent SDQ impact	0.19 (−0.25 to 0.62)	0.93 (0.51–1.67)	0.28 (−0.15 to 0.72)	1.06 (0.62–1.81)	0.03 (−0.39 to 0.45)	0.81 (0.42–1.54)
Youth SDQ total score	0.50 (0.05–0.94)*	2.57 (1.32–5.01)**	0.42 (−0.01 to 0.85)	2.53 (1.38–4.64)**	0.83 (−0.33 to 0.49)	1.04 (0.59−1.83)
Youth SDQ impact	0.13 (−0.30 to 0.56)	1.17 (0.63–2.17)	0.54 (0.11−0.97)*	1.26 (0.75–2.13)	−0.12 (−0.53 to 0.29)	0.70 (0.36–1.35)
SDQ subscales						
Parent SDQ emotion problems	–	–	0.54 (0.10–0.97)*	1.97 (1.08–3.58)*	–	–
Youth SDQ emotion problems	–	–	0.91 (0.44–1.38)***	5.49 (2.39–12.59)***	–	–
SDQ subscales						
Parent SDQ behaviour problems	–	–	–	–	1.85 (1.30–2.41)***	6.22 (2.53–15.27)***
Youth SDQ behaviour problems	–	–	–	–	0.64 (0.19–1.09)*	1.36 (0.71–2.59)

Age and male gender was included as covariates in the analyses

*SDQ* Strengths and Difficulties Questionnaire, *DAWBA* Development and Well-being Assessment, *OR* odds ratio

* Significance (two sided), p < .05, ** significance (two sided), p < .01, *** significance (two sided), p < .001

Findings based on supplemental analyses in the clinic samples for specific problems/disorders are presented in Additional file 2: Table S1. The parent SDQ total score was related to hyperactivity problems, conduct problems and disorders, and oppositional problems and disorders, whereas the youth SDQ total score was not related to any of these scales. Neither the parent nor the youth SDQ impact scale was associated with any of these problems/disorders. The parent SDQ hyperactivity scale was related to hyperactivity problems and disorders and the parent SDQ behaviour problems was related to conduct problems and disorders as well as to oppositional defiant problems and disorders. The youth SDQ behaviour problems scale was associated with conduct problems only.

**Fig. 1:**
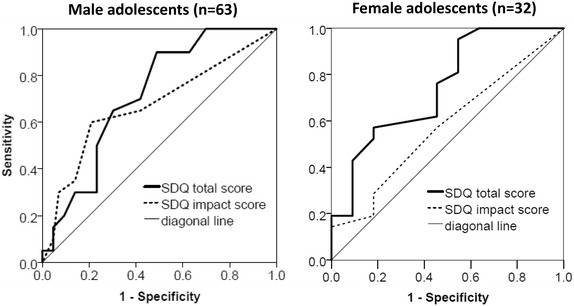
Receiver operating characteristic analyses of the SDQ total and impact score to predict emotional disorders in male and female adolescents in the clinic sample (N = 95). *SDQ* Strengths and Difficulties Questionnaire

Finally, additional ROC analyses (with the area under the curve (AUC) as a measure of diagnostic accuracy) in the clinic sample found that both the SDQ total (AUC 0.71, 95% CI 0.59–0.84, p = 0.004) and the impact score (AUC 0.67, 95% CI 0.52–0.83, p = 0.025) were significantly associated with emotional disorder in male youth. Interestingly, the SDQ impact score had higher sensitivity values whereas the total score had higher specificity values (see Fig. 1). In female youth, only the SDQ total score (AUC 0.75, 95% CI 0.56–0.93, p = 0.024) but not the impact score (AUC 0.58, 95% CI 0.37–0.78, p = 0.487) was significantly related to emotional disorders.

## Discussion

The current study adds to previous findings on the validity of multi-informant assessments of mental disorders in youth [5, 19]. Unlike earlier studies, the present investigation is based on internet-based instruments only. The DAWBA has previously been used to identify mental health disorders with similar properties to traditional diagnostic interviews such as the Diagnostic Interview Schedule for Children (DISC) and the Child and Adolescent Psychiatric Assessment (CAPA) [44]. However, the DAWBA was a more conservative measure, generating fewer diagnoses than the other two measures [44]. In the present study, two different approaches to validation were used in parallel across multiple mental health domains: First, validation against an empirically derived computerized algorithm (the DAWBA bands) and, secondly, validation against ICD-10 diagnoses by clinical experts. Overall, the two validation approaches generated similar results supporting the likely robustness of the findings. Based on the rather low prevalence rates of affective and anxiety disorders, the corresponding correlations of DAWBA bands and expert ratings were only modest in the community sample. This finding may also reflect the rather moderate agreement of different diagnostic approaches when assessing affective and anxiety disorders in youth [45]. Correlation coefficients between parent and youth SDQ scales were similar to findings from previous studies [6, 7]. However, the correlations between all reported subscales were highly significant in the clinical sample, but the total score was not. There is no clear and easy explanation to this sample-dependent finding that is in need of more detailed studies. Furthermore and in contrast to our and previous findings in community samples [34], youth and parents in the clinic sample did not agree on the level of distress and impairment caused by mental health problems. Also this finding needs further studies aiming at some clarification of the origins of these discrepant views.

### Parent and youth information to identify any mental health problems/disorders

Our findings confirmed and expanded previous findings on informant validity in both community and clinical samples of youth, [e.g., 22, 46]. In line with previous research and in agreement with hypothesis 1, we found that both the youth and parent SDQ total scores were associated with any problems/disorders in both samples. Parent and youth information is valuable for identifying mental health problems in adolescents. Each category of informant made its own unique and valuable contribution to the prediction of mental health problems in both community and clinical settings. Therefore, researchers and clinicians are strongly recommended to collect information from both youth and parents whenever possible for assessing mental health problems [19], though parent reports alone are sometimes a reasonable substitute for screening purposes when it would be impractical or unaffordable to collect information from multiple informants.

### Parent and youth information to identify emotional problems/disorders

Also in agreement with previous research and in confirming hypothesis 2, we found SDQ self reports more strongly associated with emotional problems. Youth self-reports are the best source for identifying emotional problems such as depression and anxiety in adolescents. The superiority of self-reports was independent of sample characteristic and therefore may apply for researchers assessing prevalence rates in the community as well as for practitioners in psychiatric institutions. One of the reasons is that parents may have limited access to youth’s intrapsychic processes. [26]. The superiority of self-report may not apply to younger children under the age of 11, who may not have the ability to describe their emotional problems. Furthermore, our results as well as findings of previous research show that parent information can still significantly add value for diagnostic decision making and problem description [17, 20]. Future screening instruments may use different sets of items for parent and youth to address internalizing disorders. Parent scales should specifically focus on observable behaviours that are associated with depression and anxiety (e.g., social isolation, avoidance behaviours).

### Parent and youth information to identify behavioural problems/disorders

Independent of the setting (clinical vs. community sample), we found parent reports better suited than youth self-reports for identifying behavioural problems/disorders and specifically for CD and ODD in adolescents. According to hypothesis 4, our findings confirm results of previous studies based on clinical settings that adolescent self-report show limited value for assessing ADHD [46, 47], CD [48], and ODD [32, 49]. Although some studies have previously found higher correlations between parent and youth reports for externalizing disorders [5–7, 19] and that self-reports can discriminate youth referred for conduct disorder from normal controls [50], our findings show limited additional value resulting from including self-reports to detect externalizing mental health problems in both the community and clinical samples. In clinical settings, youth may minimize problems to gain favorable reports from their clinicians. Some youth may be repressing and denying their behavioral problems or providing socially desirable responses in questionnaires [33]. In community samples, self-reports have previously been found useful in screening for externalizing disorders [20, 28–31]. Our results do not confirm these findings and hypothesis 3 and are in keeping with a clinical body of opinion that adolescent information only is not sufficient to decide on behaviour problems/disorders. Furthermore, and supporting the need for multi-informant data, parent-reported behavior problems in community youth outperformed adolescent self-reports in the prediction of later criminal outcomes in adolescence and adulthood [31]. However, given the limited sample size and the low prevalence of behaviour disorders/problems in our community study, the present findings should be treated with caution.

### The value of impact measures for identifying mental health problems/disorders

Most previous studies have focused on the presence of mental health symptoms only, rather than on how these symptoms influence individual, family and school functioning [34]. The present findings support the relevance of the youth SDQ impact score for detecting emotional problems in male adolescents in clinical settings and for detecting mental health problems/disorders in community youth. Some youth may report subclinical levels of symptoms but still report distress and impairments caused by these problems. Previous research found subclinical symptoms of adolescent depression to have long term negative effects in adulthood [51]. Our findings may indicate that the SDQ impact scale is useful for screening of early mental health problems. Our additional ROC analyses provided some indication of gender-specific differences in the identification of emotional disorders in the clinic sample. Anxious or depressed males who do not report much by way of emotional symptoms may nevertheless be aware that their life is impaired. If clinicians ask about such impairment and follow up with sensitive probing about emotional symptoms, this might improve the recognition of anxiety and depression, particularly in males.

### Strengths and limitations

This is the first study that has tested parent and youth screening measures comprehensively across multiple mental health domains simultaneously in clinical and community settings with two complementary approaches to validation (empirically validated computer algorithms and diagnoses by expert clinicians). It is reassuring that the results of the two approaches converge, supporting informant-specific assessment of psychopathology in youth. Nevertheless, the present findings have to be interpreted under the view of some limitations: First, because of the moderate sample size of the clinic sample and the low prevalence of some disorders, the statistical power for the regression analyses was limited. We therefore only provided analyses for the most frequent disorders. Secondly, the present findings were limited to the SDQ as predictor and the DAWBA as outcome. No further screening measures of psychopathology were used in the present study. Thirdly, no teacher ratings were available and could therefore not be included as further informants in this study. Forthly, because the community sample was based on European ethnicities, the findings may not generalize to other ethnic groups. Finally, family background variables (e.g., socio-economic status or parental separation) were not available and could not have been controlled for in the present study. Further studies are needed to elucidate the underlying mechanisms of discrepancies of informant validity.

## Conclusions

The current findings illustrate the importance of considering motivation and the nature of behavioural and emotional problems in self-report ratings. Clinical practitioners should keep in mind that adolescents may display problem behaviours only in specific settings but also have limited ability to report behavioural and hyperactivity problems. The “Operations Triad Model” [OTM; 5, 10] is a conceptual frame-work on how to use and interpret multi-informant assessments which is guided by evidence based information on the divergence and convergence of informants’ reports. OTM guides clinicians (a) to hypothesize about patterns of convergence and divergence among informants reports and (b) to develop personalized assessments that directly test these hypotheses. To do this, practitioners may rely on information on the context in which the problems emerge as well as the informant’s ability to report mental health problems across different domains. The current findings may guide clinicians to choose which kind of information should be collected from which informants and how to aggregate that information in order to decide on further assessment and treatment.

## Additional files

**Additional file 1:** Data of the clinic and community sample.

**Additional file 2:** **Table S1.** Ordinal and logistic regressions with SDQ parent and youth measures as predictors of specific DAWBA bands/expert diagnosis in the clinical sample (N = 95).

## Abbreviations

SDQ: Strength and Difficulties Questionnaire
DAWBA: Development and Well Being Assessment
ICD-10: International Classification of Diseases, Tenth Edition
ADHD: attention deficit hyperactivity disorders
CD: conduct disorders
ODD: oppositional defiant disorders
ASEBA: Achenbach Systems of Empirically Based Assessments
DSM-5: Diagnostic and Statistical Manual of Mental Disorders, Fifth Edition
SD: standard deviation
ROC: receiver operating characteristic
DSM-IV: Diagnostic and Statistical Manual of Mental Disorders, Forth Edition
SPSS: Statistic Package for Social Scientists
AUC: area under the curve
