# Supplementary material for: The contribution of parent and youth information to identify mental health disorders or problems in adolescents
Source: Child Adolesc Psychiatry Ment Health. 2017 Apr 28;11:23. doi: 10.1186/s13034-017-0160-9 (PMC5408828; doi:10.1186/s13034-017-0160-9)
Supplement: Supplementary file 2 — Additional file 2: Table S1. Ordinal and logistic regressions with SDQ parent and youth measures as predictors of specific DAWBA bands/expert diagnosis in the clinical sample (N = 95). [file 13034_2017_160_MOESM2_ESM.docx]

**Supplemental table S1:**  Ordinal and logistic regressions with SDQ parent and youth measures as predictors of specific DAWBA bands/expert diagnosis in the clinical sample (N = 95)

| DAWBA bands | Hyperactivity problem/disorder | | Conduct problem/disorder | | Oppositional defiant problem/disorder | |
| --- | --- | --- | --- | --- | --- | --- |
| Variables | DAWBA band  Estimate (95%CI) | Expert Diagn.  OR (95%CI) | DAWBA band  Estimate (95%CI) | Expert Diagn.  OR (95%CI) | DAWBA band  Estimate (95%CI) | Expert Diagn.  OR (95%CI) |
| SDQ total/impact score |  |  |  |  |  |  |
| Parent SDQ total score | **1.09 (0.60-1.58)***** | 2.19 (0.99-4.85) | **0.73 (0.27-1.19)**** | **3.48 (1.13-10.80)*** | **0.75 (0.31-1.18)**** | **2.21 (1.07-4.57)*** |
| Parent SDQ impact | 0.32 (-0.12-0.76) | 1.35 (0.62-2.97) | -0.08 (-0.52-0.36) | 0.83 (0.29-2.40) | -0.02 (-0.44-0.40) | 0.66 (0.31-1.42) |
| Youth SDQ total score | -0.04 (-0.46-0.39) | 1.05 (0.45-2.49) | 0.27 (-0.17-0.71) | 2.40 (0.79-7.25) | -0.00 (-0.41-0.40) | 0.96 (0.47-2.00) |
| Youth SDQ impact | -0.04 (-0.47-0.39) | 0.78 (0.32-1.95) | -0.15 (-0-58-0.28) | 0.22 (0.04-1.35) | -0.02 (-0.42-0.39) | 1.02 (0.50-2.10) |
| SDQ subscales |  |  |  |  |  |  |
| Parent SDQ hyperactivity | **1.76 (1.18-2.33)***** | **5.21 (1.78-15.25)***** | -- | -- | -- | -- |
| Youth SDQ hyperactivity | 0.20 (-0.25-0.64) | 1.24 (0.54-2.84) | -- | -- | -- | -- |
| SDQ subscales |  |  |  |  |  |  |
| Parent SDQ behavior problems | -- | -- | **1.26 (0.77-1.76)***** | **3.99 (1.40-11.35)**** | **1.33 (0.85-1.80)***** | **2.65 (1.25-5.65)*** |
| Youth SDQ behavior problems | -- | -- | **0.57 (0.12-1.01)*** | 1.15 (0.46-2.66) | 0.41 (-0.01-0.82) | 1.51 (0.78-2.84) |

**Note**. SDQ= Strengths and Difficulties Questionnaire, DAWBA= Development and Well-being Assessment. OR=odds ratio. * = significance (two sided), p < .05, ** = significance (two sided), p < .01, *** = significance (two sided), p < .001. Age and male gender was included as covariates in the analyses.
